# Transposable elements activation triggers necroptosis in mouse embryonic stem cells

**DOI:** 10.1038/s41419-023-05705-3

**Published:** 2023-03-07

**Authors:** Lingmei Jin, Jiangping He, Huijian Feng, Sa Li, He Liu, Hongzhi Dong, MingLi Hu, Junju Huang, Haoyu Wu, Jiekai Chen, Ling Qi, Kaixin Wu

**Affiliations:** 1 Institute of Digestive Disease, the Sixth Affiliated Hospital of Guangzhou Medical University, Qingyuan People’s Hospital, B24 Yinquan South Road, Qingyuan, 511518 Guang Dong China; 2 CAS Key Laboratory of Regenerative Biology, Guangdong Provincial Key Laboratory of Stem Cell and Regenerative Medicine, Guangzhou Institutes of Biomedicine and Health, Chinese Academy of Sciences, Guangzhou, 510530 China; 3 Center for Cell Lineage and Atlas (CCLA), Bioland Laboratory, Guangzhou Regenerative Medicine and Health GuangDong Laboratory, Guangzhou, China; 4 Guangzhou Laboratory, Guangzhou, 510005 Guangdong Province China; 5 University of Chinese Academy of Sciences, Beijing, 100049 China; 6 Centre for Regenerative Medicine and Health, Hong Kong Institute of Science & Innovation, Chinese Academy of Sciences, Hong Kong SAR, China; 7 Department of Developmental Biology, School of Basic Medical Sciences, Southern Medical University, Guangzhou, China

**Keywords:** Embryonic stem cells, Gene silencing, Necroptosis

## Abstract

Deficiency of the histone H3K9 methyltransferase SETDB1 induces RIPK3-dependent necroptosis in mouse embryonic stem cells (mESCs). However, how necroptosis pathway is activated in this process remains elusive. Here we report that the reactivation of transposable elements (TEs) upon SETDB1 knockout is responsible for the RIPK3 regulation through both *cis* and *trans* mechanisms. IAPLTR2_Mm and MMERVK10c-int, both of which are suppressed by SETDB1-dependent H3K9me3, act as enhancer-like cis-regulatory elements and their RIPK3 nearby members enhance RIPK3 expression when SETDB1 is knockout. Moreover, reactivated endogenous retroviruses generate excessive viral mimicry, which promotes necroptosis mainly through Z-DNA-binding protein 1 (ZBP1). These results indicate TEs play an important role in regulating necroptosis.

## Introduction

Transposable elements including endogenous retroviruses (ERVs) occupy surprisingly high components of the mammalian genome [1]. TEs were initially regarded as useless DNA sequences in the genome. Whereas, more and more evidence showed that TEs can participate in the normal life process, and their activation may cause genome instability and diseases [2]. Many integrated TEs have become parts of host cell genes (e.g., RAG enzymes) [3]. TEs can self-replicate in the genome and they are coopted to form new regulatory elements such as enhancers, promoters, and insulators. Therefore, TEs can regulate gene expression during evolution, and contribute to the rewiring of gene regulatory networks [4–6]. Activated TEs can be transcribed to produce RNAs such as long non-coding RNAs (lncRNAs), microRNAs and circular RNAs. Among them, 83% of human lncRNAs contains TEs and 42% of lncRNAs is derived from TEs [1]. Primate-specific ERVs such as HERV-H, are closely involved in the pluripotency regulatory network in human embryonic stem cells. Knockdown of the lncRNAs transcribed from HERV-H can lead to rapid differentiation of human embryonic stem cells [7]. ERVs are orchestrally activated in the early embryonic development, meanwhile SETDB1, KAP1, G9A, DNMT3A, etc., sequentially play an important role in the process [8]. Many regulatory elements of pluripotency genes such as *Pou5f1* and *Nanog* fall into the ERVs [9]. These results indicate that TEs play an important role in the early embryonic cell fate determination.

Usually, TEs are silenced by DNA methylation in somatic cells [10]. However, the genome is globally hypomethylated in the early embryonic developmental stage such as in the blastocyst, and TEs are suppressed by a zinc-finger proteins (ZFPs)-TRIM28 with context-specific patterns of chromatin marks [11]. The H3K9 methyltransferase SETDB1 interacts with TRIM28 and plays a crucial role in the silencing of type I and type II ERVs [9, 12, 13]. Depletion of SETDB1 activates a large number of ERVs including MERVL, a two-cell (2 C) like state marker. Our previous studies have shown that loss of SETDB1 induces pluripotent state to 2C-like state transition in serum/LIF (SL) condition, and rapid cell death in the form of necroptosis by activating RIPK3 in the two inhibitors (2i) and LIF (2iL) condition [14]. However, the underlying mechanism of how *Ripk3* is activated remains unclear.

Necroptosis is a type of programmed cell death that differs from apoptosis and traditional necrosis and is related to many biological processes, such as tumorigenesis, pathogen invasions and inflammatory diseases [15–17]. It is initiated by the regulation of tumor necrosis factor receptors or pattern recognition receptors (PRRs) [18–20]. Furthermore, tumor necrosis factor ɑ (TNFɑ) and exogenous nucleic acid from pathogen can activate RIPK3 and induce immune response through PRRs such as Toll-like receptor3 (TLR3) and Z-DNA binding protein 1 (ZBP1) [21]. Formation of necrosome induced by active phosphorylated RIPK3/RIPK1 is the hallmark of necroptosis [22]. It is possible that expression of TEs could mimic virus invasion to induce necroptosis through PPRs activation pathway.

Here, we demonstrate that TEs induce the activation of RIPK3 to trigger necroptosis in SETDB1-KO mESCs under 2iL condition. On the one hand, TEs (i.e., IAPLTR2_Mm, MMERVK10c-int) function as enhancers to regulate gene expression such as *Ripk3*. Moreover, TE activation produces double strand RNA (dsRNA), which is recognized by ZBP1 and then activates RIPK3 to trigger necroptosis. These data reveal that the activation of TEs leads to necroptosis in mESCs, highlighting the important role of TEs in life process.

## Results

### Gene activation is associated with reactivated TEs in SETDB1 depletion mESCs

**Fig. 1:**
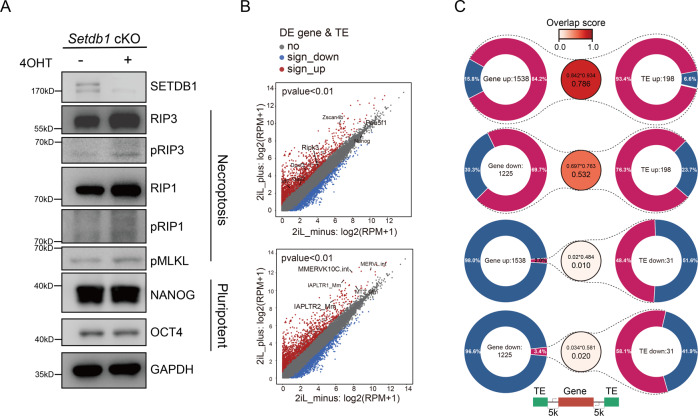
Depletion of *Setdb1* activates necroptosis and a large number of of TEs. **A** Western blot analysis of the necroptotic markers such as phosphorylation RIP3, phosphorylation RIP, phosphorylation MLKL and pluripotent markers such as NANOG and OCT4 in *Setdb1* cKO cell with/without 4OHT treatment. **B** Volcano plots of up-regulated and down-regulated genes and TEs after *Setdb1* knockout in 2iL condition. **C** Pie chart of the up-regulated/down-regulated genes nearby TEs (5 kb). Overlap score range from 0 to 1.

**Fig. 2:**
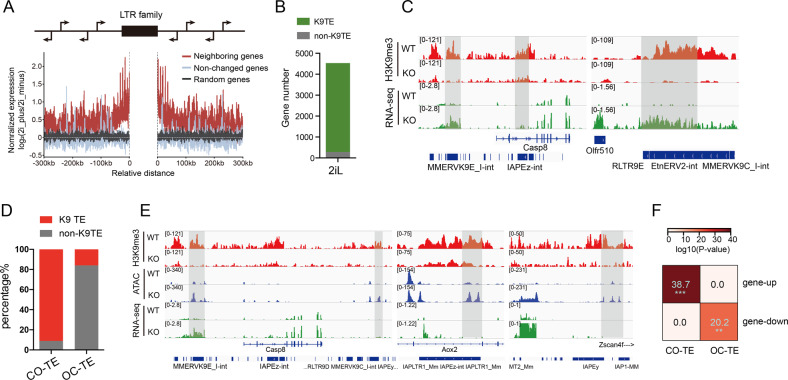
H3K9me3 marked TEs display a cis-regulatory effect. **A** Line plot showing the expression trends of neighboring genes within 300 kb of LTR family, expression non-changed genes and random genes act as a control. **B** Histogram displays gene number nearby H3K9me3 marked-TEs and non-H3K9me3-marked TEs in 2iL condition. **C** IGV graphs illustrate H3K9me3 occupancy in *Setdb1* WT and KO mESCs in 2iL culturing condition, together with RNA-seq in 2iL culturing conditions at *Mmp10* and *Olfr510* with their nearby TEs. **D** Column graphs shows percentage of K9/non-K9-marked TEs in CO/OC-TE. **E** IGV graphs illustrate H3K9me3 occupancy in *Setdb1* WT and KO mESCs in 2iL culturing condition, together with RNA-seq and ATAC-seq in 2iL condition at *Casp8*, *Aox2*, *Zscna4f* with their nearby TEs. **F** A heatmap displays CO/OC-TEs and their nearby up/down regulated genes. Significance is derived from a Fisher exact test (*p < e-10; **p < e-20; ***p < e-30).

SETDB1 knockout cause necroptosis with *Ripk3* activation and maintained pluripotency state in 2iL culture mESCs (Fig. 1A). Specifically, RIP1 and RIP3 interacted in *Setdb1* knockout cells under 2iL condition, and the necroptosis can be inhibited by RIP1 inhibitor Nec-1 (Fig. S1A, B). Previous study has shown the essential role of SETDB1 in suppressing ERVs cooperating with KAP1 in mESCs [13] (Fig. S2A). Depletion of SETDB1 activated genes related to necroptosis as well as a large number of ERVs including intracisternal A particle (IAP) family (Fig. 1B). Surprisingly, we found that activated genes were always accompanied by activated TEs, indicating TEs may act as cis regulatory elements (Fig. 1C). It was reported that long terminal repeat elements (LTR elements) such as ERVs have been evolved in functioning as host promoter, enhancer, and insulator elements to establish gene regulatory networks [4] (Fig. S2A). Specially, genes nearby LTR family are more likely to be activated after SETDB1 depletion, and most of them are H3K9me3 located TEs (K9TE) (Fig. 2A–C). As H3K9me3 is a heterochromatin marker, its removal may change chromatin accessibility. Therefore, we carried out ATAC sequencing and found that the chromatin state of most K9TEs shifted from close to open (represented by “CO”) and non-K9TE preferred being open than close (represented by “OC”) (Fig. 2D). We observed several examples of up-regulated genes (e.g., *Casp8*, *Zcan4f*) were accompanied with chromatin open K9TE after SETDB1 KO (Fig. 2E). Further analysis showed that CO-TE were enriched up-regulated genes and OC-TE were enriched down-regulated genes (Fig. 2F). These results indicated that H3K9me3-marked TE has cis regulatory effect on nearby genes.

### IAP elements are enriched in *Setdb1*-KO mediated necroptosis

**Fig. 3:**
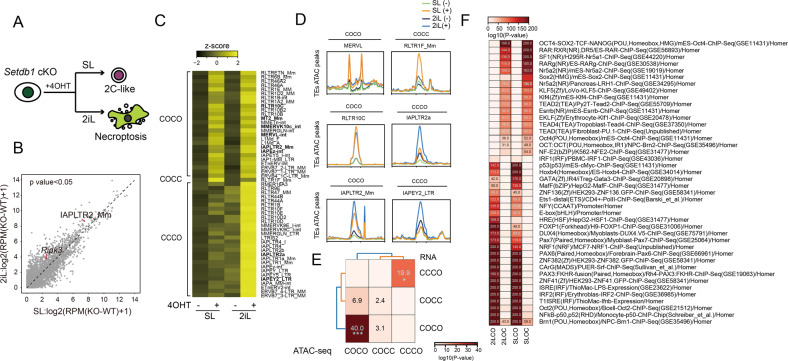
IAP elements are enriched in *Setdb1*-KO mediated necroptosis related TEs enrich. **A** Cell fate model of *Setdb1* KO mESCs under SL and 2iL conditions. **B** Volcano plot of upregulated genes and TEs in 2iL and SL culturing conditions. **C** Heatmap profile from ATAC-seq analysis classifies TE expression of *Setdb1* CKO cells upon SL or 2iL condition into 3 clusters (COCO,COCC and CCCO). **D** Read-count tag density pileups of ATAC profiles on representative TEs in cluster “COCO”, “COCC”,”CCCO”. **E** A heatmap displays the enrichment of ATAC-seq clusters in RNA defined clusters. Significance is derived from a Fisher exact test. **F** Heat map of different transcription factors’ motif analysis in 2iL CO/OC and SL CO/OC clusters (*p < e-10; **p < e-20; ***p < e-30).

Deficiency of SETDB1 induced 2C-like state transition in SL condition and induced necroptosis in 2iL condition (Fig. 3A). Known as the key regulator of necroptosis, *Ripk3* and its upstream IAPLTR2_Mm element both had higher activation range in 2iL condition after *Setdb1* Knockout (Fig. 3B, S1B). In our previous study, we used “0” and “1” to represent the low and high expression level of RNA, therefore, “01” and “10” mark up- and down-regulation, respectively [14]. Here, by comparing the ATAC peaks at each locus after *Setdb1* knockout in SL/2iL condition, we divided TEs into several clusters according to their chromatin accessibility patterns, in which cluster “COCO” represents chromatin state more open in both SL and 2iL conditions upon *Setdb1*-KO, cluster “COCC” and “CCCO” represent chromatin state more open in only SL or 2iL conditions upon *Setdb1*-KO (Fig. 3C). Specifically, Many IAPs (e.g., IAPLTR2a, IAPEY2_LTR) elements were enriched in “CCCO” cluster, and “CCCO” was highly correlated with “0001” cluster (Fig. 3D, E). It indicated that IAP family may play a role in *Setdb1*-KO mediated necroptosis. Furthermore, analysis of ATAC showed that closed chromatin was enriched with pluripotency related motif and open chromatin was enriched with immune related motif (Fig. 3F). Correspondingly, Those H3K9me3-dependent ERVs (e.g., IAPLTR1_Mm, LTRIS2, MMERVK10c-int) marked “0001” and “0101” genes were enriched in immune response and TRAIL-activated apoptotic signaling pathways (Fig. S2C–E).

### ERV elements IAPLTR2_Mm and MMERVK10c-int function as cis-regulatory elements in regulating *Ripk3* expression

**Fig. 4:**
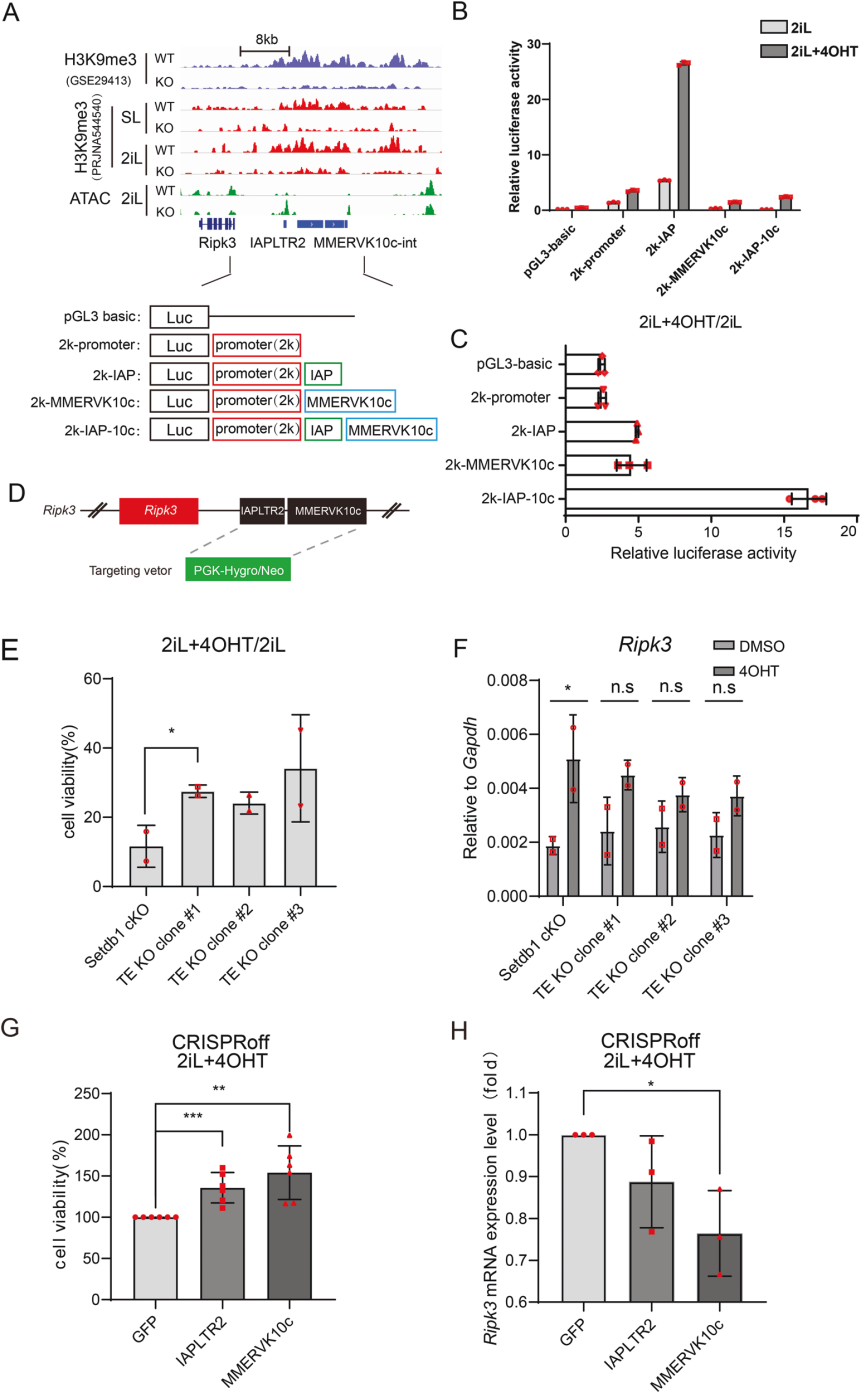
*Ripk3* expression was regulated by its upstream ERVs. **A** IGV graphs illustrate H3K9me3 occupancy ± 4OHT treatment in SL and 2iL culturing conditions, together with ATAC-seq in 2iL culturing conditions at *Ripk3* and its upstream ERVs loci. Dual luciferase reporter system plasmids were constructed according to the IGV graphs. **B**, **C** Column graphs summarize the luciferase activity of *Setdb1* cKO cells transfected by luciferase reporter system plasmids with different combinations of *Ripk3* promoter, IAPLTR2_Mm and MMERVK10c-int. *n* = 3, technical replicates. **D** Schematic view of the upstream TE of *Ripk3* KO strategy. **E** Cell viability assay of WT and TE KO cells of *Setdb1* CKO mESCs treated with/without 4OHT using CCK8 test (*n* = 2 biological replicates; error bar, SD; unpaired *t*-test, *p < 0.05). **F** qPCR analysis of *Ripk3* expression in Setdb1 cKO cells and TE KO cells with/without 4OHT treatment. (*n* = 2, biological replicates; error bar, SD; *t*-test, *p < 0.05). **G** Cell viability assay of *Setdb1* cKO cells overexpressing CRISPRoff using CCK8 test. Cells were transfected with control sgRNA and sgRNA targeting IAPLTR2 and MMERVK10c. (*n* = 6 biological replicates; error bar, SD; unpaired *t*-test, *p < 0.05, **p < 0.01, ***p < 0.001). **H** *Ripk3* expression of *Setdb1* cKO cells overexpressing CRISPRoff. Cells were transfected with control sgRNA and sgRNA targeting IAPLTR2 and MMERVK10c. (*n* = 3 biological replicates; error bar, SD; unpaired *t*-test, *p < 0.05, **p < 0.01, ***p < 0.001).

RIPK3 is a member of the receptor-interacting protein (RIP) family of serine/threonine protein kinases and is one of the core components of necroptosis. The upstream sequence of *Ripk3* is marked with *Setdb1*-dependent H3K9me3, centering on IAPLTR2_Mm and MMERVK10c-int elements (Fig. 4A). As LTR elements has cis-regulatory function and consistent with the previous finding, our ATAC-seq data showed increased chromatin accessibility on IAPLTR2_Mm of *Ripk3* after SETDB1 knockout under 2iL condition, suggesting IAPLTR2_Mm may function as an enhancer (Fig. 4A). To test whether IAPLTR2_Mm and MMERVK10c-int elements have regulatory effects on *Ripk3* expression, we constructed dual luciferase reporter plasmids with different combinations of *Ripk3* promoter, IAPLTR2_Mm and MMERVK10c-int (Fig. 4A). We found that together with *Ripk3* promoter, IAPLTR2_Mm promotes luciferase activity, while MMERVK10c-int inhibits luciferase activity in 2iL cultured mESCs (Fig. 4B, C). In combination with *Ripk3* promoter, both IAPLTR2_Mm and MMERVK10c-int were activated in 2iL upon *Setdb1*-KO and *Ripk3* promoter combined with both IAPLTR2_Mm and MMERVK10c-int owned the highest activation degree (Fig. 4C). Further experiments showed that deletion of IAPLTR2_Mm and MMERVK10c-int elements at the upstream of *Ripk3* weakened *Ripk3* expression and rescued cell viability in the *Setdb1* knockout ES cells (Fig. 4D–F). Moreover, we used dCas9 system to inhibit IAPLTR2 and MMERVK10c-int expression (Fig. S3A). It showed that inhibition of IAPLTR2 and MMERVK10c-int by CRISPRoff system rescued cell viability (Fig. 4G) [23]. Meanwhile, effective TE inhibition resulted in the decrease of *Ripk3* expression (Fig. 4H). These results suggested that TEs act as cis-regulatory elements to regulate *Ripk3* expression in the absence of SETDB1-H3K9me3 repression.

### ERVs activation mimic virus invasion to induce necroptosis

**Fig. 5:**
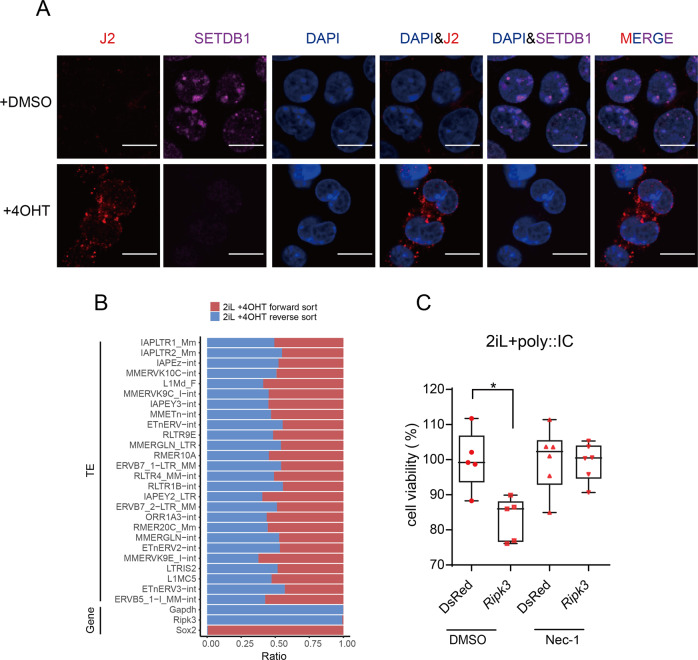
ERVs activation mimic virus invasion to induce necroptosis. **A** Immunofluorescence of J2 and SETDB1 in *Setdb1* cKO cells treated with DMSO or 4OHT. Scale bars, 10 μm. **B** Heatmap shows transcription direction of TEs and genes analyzed from strand-specific RNA-Seq. **C** Cell viability assay of *Setdb1* cKO cells overexpressing dsRed/*Ripk3* transfected by poly(I:C) using CCK8 test. Cells were treated with DMSO or Nec-1. (*n* = 6, 2 biological replicates; error bar, SD; *p < 0.05).

Necroptosis can be initiated by many stimuli including dsRNA stimuli [18]. It is reported that LINEs and ERVs exhibit bidirectional transcription and can pair to form dsRNA [24, 25]. J2 immunofluorescence displayed the production of dsRNA upon *Setdb1* KO (Fig. 5A). We next performed strand-specific RNA-seq and analyzed the bidirectional transcription of TEs upon SETDB1 depletion. We found that the activated LINEs and ERVs upon SETDB1 knockout exhibited sense and antisense transcription while genes showed single directional transcription (Fig. 5B). The TE regions predicted to generate dsRNAs were shown on the RNA-seq density plots (Fig. S4A). And virus mimetic polyinosinic: polycytidylic acid (polyI:C) induced necroptosis under *Ripk3* overexpression, confirming the effect of ERVs activation in inducing necroptosis (Fig. 5C).

### ZBP1 senses ERVs to trigger necroptosis under SETDB1 deficiency

**Fig. 6:**
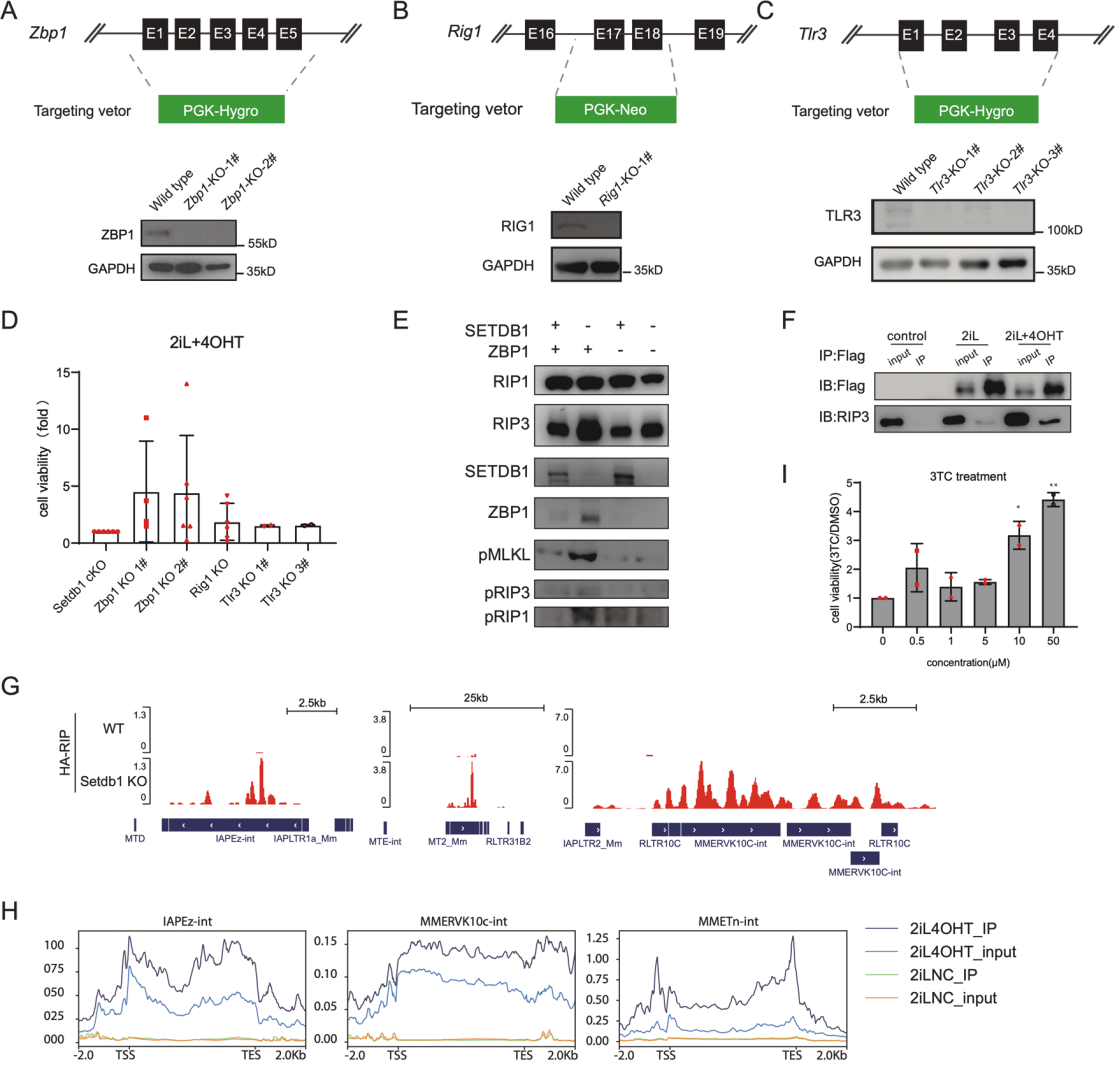
ZBP1 senses ERV RNA/DNA to trigger necroptosis. **A**–**C** Schematic view of Zbp1/Rig1/Tlr3 KO strategy and western blot analysis demonstrates the expression of ZBP1/RIG1/TLR3 in wild type and corresponding knockout mESCs. **D** Cell viability assay of *Zbp1* KO, *Rig1* KO and *Tlr3* KO cells using CCK8 test. (*n* = 4, biological replicates; error bar, SD; *p < 0.05, **p < 0.01) **E** Western blot analysis of the necroptotic markers such as RIP3 and pRIP in *Setdb1* cKO cell and *Zbp1* KO cells with/without 4OHT treatment. **F** Co-IP of overexpressed 3×Flag-HA-ZBP1 and RIPK3. **G** IGV of HA-RIP on IAPEz-int, MT2 and MMERVK10c. **H** Representative ERVs enriched by 3×Flag-HA-ZBP1. **I** Cell viability assay of Setdb1 cKO mESCs treated with different concentration of 3TC (*n* = 2, biological replicates; error bar, SD; *p < 0.05, **p < 0.01).

Intracellular dsRNAs are recognized by pattern recognition receptors including TLR3, MDA5 (encoded by *Ifih1*), and RIG-I (encoded by *Ddx58*) [26], among which ZBP1 was reported to sense dsRNA to trigger necroptosis in gut stem cell [27]. We analyzed the expression pattern of DNA/RNA sensor after *Setdb1* KO and found that *Ddx58*, *Tlr3,* and *Zbp1* were significantly activated in 2iL condition following “0001” expression pattern after *Setdb1* KO (Fig. S5A). To further investigate the key role of RIG-1, TLR3 and ZBP1 in TEs induced necroptosis, we knocked out *Zbp1*, *Rig1* and *Tlr3* respectively, and validated the deletion from both mRNA and protein levels (Fig. 6A–C, Fig. S5B). We observed that ZBP1 knockout could partially rescued *Setdb1*-KO induced necroptosis while RIG-I and TLR3 knockout showed little effect (Fig. 6D). Correspondingly, phosphorylation of RIP1, MLKL, and RIP3 were decreased after ZBP1 KO upon *Setdb1* KO in 2iL condition (Fig. 6E). ZBP1 can interact with RIP3 upon *Setdb1* KO in 2iL condition and bind several activated ERVs such as IAPEz-int, MT2_MM and MMERVK10C-int (Fig. 6F–H, Fig. S4C). As ZBP1 recognized Z-DNA/Z-RNA and RNA transcript from retrotransposons can retrotranscript into cDNA and inserted into the genome [28, 29], we next tested RTase inhibitor (TRi) and found 3TC could rescue *Setdb1*-KO induced necroptosis (Fig. 6I). These results suggested that transcripts from activated TEs were recognized by ZBP1 that further triggers necroptosis under SETDB1 deficiency.

## Discussion

In the present study, we report that activation of TEs is responsible for necroptosis in the SETDB1 knockout mESC. Our previous studies have shown that SETDB1 knockout mediated necroptosis under 2iL condition but the underlying mechanism was unknown [14]. TEs invasion have catalyzed the evolution of gene-regulatory networks and participated in life activities [30, 31]. SETDB1 repress TEs in mESC [13]. We confirmed that IAPLTR2_Mm and MMERVK10c-int function as cis regulatory element to regulate expression of the nearby gene *Ripk3* (Fig. 4A–C). However, inhibition of *Ripk3* or deletion of upstream IAPLTR2_Mm and MMERVK10c-int sequences only partially rescue cell viability under SETDB1 knockout (Fig. 4F) [14], suggesting that there are other TEs or mechanisms involved in SETDB1-KO mediated cell death. H3K9me3-dependent TE located “0001” and “0101” gene clusters are enriched in immune response, aging, and TRAIL-activated apoptotic signaling pathways (Fig. S2E). It suggested that TEs activation triggered cell death upon SETDB1-KO in several other pathways (e.g., immune response, aging, and TRAIL-activated apoptotic signaling pathways) besides necroptosis.

In addition to cis-regulatory elements contributing and transcription networks modifying, TEs may trigger innate immune response by its RNA transcripts and TE-derived extra-chromosomal DNA copies [32]. Activation of TEs exhibited bidirectional transcription and paired to form viral mimicry dsRNAs, which may stimulate PRRs to trigger downstream immune response [18, 24, 33]. Toll-like receptors (TLRs) (e.g., TLR3/7/8), RIG-I-like receptors (RLRs)(e.g., LGP2, RIG-I, and MDA5), OAS-like receptors (OLRs), NOD-like receptors (NLRs), and RNA-dependent protein kinase PKR are the five major types of RNA sensing pattern recognition receptors in mammalian cells [34]. Activation of TLRs and RLRs leads to cytoplasmic signaling cascades converging on activation of three major transcriptional factors—NFkB, AP1, and IRF3/7, which respond to pro-inflammatory and Type I IFNs and death pathways [35]. Therefore, PRRs may be the downstream response factor of TEs activation. Virus mimetic polyI:C induced necroptosis with *Ripk3* overexpression, which confirmed the trans regulatory effect of TEs (Fig. 5C). However, we found ZBP1 to be the effective PRRs but not RNA sensing PRR RIG-1 and TLR3 (Fig. 6E). It was reported that ZBP1 is an RHIM (RIP homotypic interaction motif) domain-containing protein, associating with RIPK1 and RIPK3 to participate in necroptosis and inflammation [36, 37]. ZBP1 is a Z-DNA/Z-RNA binding protein and a recent study found that SETDB1 dysfunction mediated reactivated TEs can activate ZBP1 for RIP3-MLKL necroptosis in gut stem cell [27]. LTRs and LINEs belonging to Class 1 elements, mobilize through a ‘copy-and-paste’ mechanism whereby a RNA intermediate is reverse-transcribed into a cDNA copy to integrate elsewhere in the genome [38]. It suggested that ZBP1 could sense both RNA and DNA derived from TEs. And RTi experiment confirmed the role of reverse transcription of TEs in Necroptosis (Fig. 6I). Besides, we are curious about whether ZBP1 is predisposed to bind specific TEs.

**Fig. 7:**
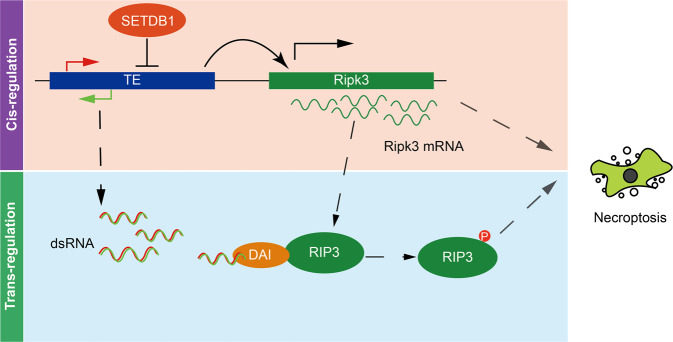
ERVs activation results in cell necroptosis through cis and trans regulation. Activation of ERVs upstream *Ripk3* enhances *Ripk3* expression and cause necroptosis. ERVs produces dsRNA and cause necroptosis through ZBP1 (DAI).

In conclusion, our study found the important role of TEs in triggering necroptosis in both cis and trans regulatory manner in *Setdb1*-KO mESC (Fig. 7). We suggest that the mechanism of SETDB1-KO mediated necroptosis with TEs activation is a general mechanism in pluripotent stem cells. And this research broadens our understanding of TEs’ function in cell fate transition and disease occurrence.

## Materials and methods

### Cell lines and cell culture condition

OG2 mouse embryonic stem cells were derived from 3.5 d.p.c ICM from 129 female mice crossing OG2 male mice. All mouse ES cell lines were grown at 37 °C under 5% CO2 on plate coated with 0.2% gelatin (homemade) in serum-free 2i/LIF media composed as follows: 1:1 mix of High glucose DMEM (SH30022.01, Hyclone) and Knockout DMEM (10829018, Gibco) with 0.5× N2 (17502048, Gibco), 0.5× B27 (17504044, Gibco), 0.1 mM 2-mercaptoethanol (M3148, Sigma), 1× GlutaMAX (35050079, Gibco), 1× MEM NEAA (11140076, Gibco), 1000U/ml LIF (homemade), 1 μM PD0325901 (DC1056, DC), and 3 μM CHIR99021 (DC1023, DC). Medium was refreshed every day and cells were passaged every 3 days using 0.05% Trypsin-EDTA (25300120, Gibco). Cells during gene editing were cultured in SL + 2i (serum LIF with 2i) medium composed as follows: Knockout DMEM with 15% FBS, 1× GlutaMAX, 1× NEAA, 0.1 mM 2-mercaptoethanol, 1000 U/ml LIF, 1 μM PD0325901, and 3 μM CHIR99021.

*Zbp1* knock out cell lines, *Rig1* knock out cell lines and *Tlr3* knock out cell lines were edited in the *Setdb1* cKO cell line using CRIPSR Cas9. We deleted exon2-4 of *Zbp1*, exon17-18 of *Rig1* and exon1-4 by homologous recombination, respectively. *Zbp1* KO cells were selected with Hygromycin for 3 days and conducted genotype identification with genome PCR, real time quantative PCR and western blot. *Rig1* KO and *Tlr3* KO cell lines were selected using the same procedure.

TE knock out cell lines were also edited in the *Setdb1* cKO cell line by CRISPR Cas9. We deleted two TEs, MMERVK10c-int and IAPLTR2_Mm upstream of *Ripk3* by homologous recombination. Cells were selected with Hygromycin and Neomycin for 5 days and conducted genotype identification with genome PCR and qPCR.

### Western blot

Western blots were performed using typical laboratory procedures. PVDF membranes were blocked using 5% non-fat milk and were incubated with primary antibodies overnight at 4 °C. After washing with TBS supplemented with 1% tween-20, membranes were incubated with HRP-conjugated polyclonal goat anti-rabbit or goat anti-mouse secondary antibodies at room tempareture for 1 h.ECL kit was used to develop the signal. Bio-rad machine were employed to acquire band images. Antibodies used in this study were listed in online appendix.

### Immunofluorescence

Cells were treated with 4OHT or DMSO for 3 days before seeded on a Matrigel coated confocal dish. After 12 h, cells were fixed in 4% paraformaldehyde for 10 min, washed with PBS for 3 times, and permeabilized with 0.5% Triton X-100 in PBS for 5 min at room temperature. Then, cells were blocked with 0.1% Triton X-100 and 5% BSA in PBS for 30 min. After blocking, cells were incubated with anti-J2 and anti-SETDB1 overnight at 4 °C. After washing with PBS, the Goat-anti-mouse/rabbit secondary antibodies (A-11004, A32733, Invitrogen) were applied for 1 h followed by DAPI (D1306, Invitrogen) staining for 2 min. Finally, the coverslips were mounted on the dishes for imaging and observation. Antibodies used in this study were listed in online appendix.

### Cell survival assay

Cell survival assay was performed using the Cell Counting Kit-8 Assay kit according to the manufacturer’s instructions (C0037, Beyotime). mESCs were pretreated with 0.5 μM 4OHT for 2 days, followed by re-plating in 96-well plates for 4 days.

### Total RNA extraction

Total RNA from cell lines was isolated using TRIZOL(MRC) method. RNA quantity and quality were assessed with NanoDrop One C spectrophotometer (ND-ONEC-W, Thermo Fisher Scientific). Only RNA with an absorbance read ratio 260/280 between 1.8–2.0 was used for experiments.

### qRT-PCR

1 ug Total RNA was used for cDNA synthesis with HiScript II Q RT SuperMix for qPCR (R222-01, Vazyme), and the diluted cDNA was used as template for qPCR with ChamQ SYBR qPCR Master Mix (Q311-02, Vazyme). Specific primers were designed for each gene transcript and are listed in Online Appendix: Reagents. All reactions were run in QuantStudio 3 Real-Time PCR System (Thermo Fisher Scientific).

### Co-immunoprecipitation (co-IP) essay

Overexpress 3×Flag-HA-ZBP1 or RIP1-HA in *Setdb1* cKO mES cells were collected with 1 × 10^7^ cells per tube. The cells were resuspended in 1 ml lysis buffer (50 mM Tris-HCl pH7.4, 1% NP-40,150 mM NaCl, 1 mM EDTA, 5% Glycerol and protease inhibitor) and rotate 1 hat 4 °C. Cell lysate was collected by centrifugation (10,000 g, 10 min at 4 °C) and incubated with 10 ul Anti-HA Magnetic beads (B26201, Selleck) or 10 ul Anti-Flag M2 beads (M8823, Millipore) for 3 h at 4 °C. Then, beads were washed with lysis buffer (without protease inhibitor) for 5 times (10 min each time) and boiled in SDS buffer for 10 min to elute the protein complex.

### HA-RNA immunoprecipitation (HA-RIP)

HA-RIP is done based on the EZ-Magna RIP kit (17-701, Millipore). Overexpress 3×Flag-HA-ZBP1 in *Setdb1* cKO cells were seeded on 15 cm dishes. Experiments are performed when adherent mammalian cells at ~80–90% confluency. Cells were overlaid with 5 ml PBS and crosslinked with 400 mJ/cm^2^ (254 nm). Cells are collected by 0.05% trypsin and washed with ice-cold PBS for twice. Discard the supernatant and resuspend cell pellet use equal volume of complete RIP Lysis Buffer. Mix by pipetting up and down until the cells have been dispersed and the mixture appears homogeneous. Incubate the lysate on ice for 5 min. Use liquid nitrogen to quick freeze the lysate and thaws the lysate quickly in room temperature. Centrifuge at 14,000 rpm for 10 min at 4 °C. Prepare the RIP Immunoprecipitation Buffer. Add 100 μl cell lysate to each tube. Then add 900 μl RIP Immunoprecipitation Buffer and 20 μl Anti-HA Magnetic beads. Incubate the tubes with rotating overnight at 4 °C. Remove 10 μl of the supernatant of RIP lysate and place it into a new tube and label “input”. Wash the beads for 6 times (5 min each time) using RIP Wash Buffer. Place the tubes on the magnetic separator and discard supernatant. Add 500 μl TRIZOL to each tubes (including input). RNA was isolated using TRIZOL(MRC) method.

### Total RNA-seq and data analysis

Total RNA-seq was performed as previously described [39]. Total RNA-seq (H/M/R) Libaray Prep Kit for Illumina (NR603, VAHTS) was used for RNA library preparation. In brief, 1 ug total RNAs were hybridized with rRNA probe (H/M/R) and digested by RNase H to remove ribosomal RNAs. After DNase I digestion, ribosomal-depleted RNAs were fragmented at 94 °C for 8 min. Then, the first-strand and second-strand cDNAs were synthesized successively using the provide reagents. cDNA was purified by VAHTS DNA Clean Beads (NR411, VAHTS), followed by end repair, dA-tailing, adaptor ligation and second-strand cDNA digestion. After two rounds of purification, the cDNAs were PCR-amplified and purified using VAHTS DNA Clean Beads.

### Luciferase assay

Upstream 2000 base pair of *Ripk3* TSS was PCR amplified and cloned upstream of firefly *luciferase* gene in the vector. *Setdb1* cKO cells were plated in 24-well plates at 2 × 10^4^ per well, and the TE /control vector (0.5 μg per well), pGL3-reporter (100 ng per well) and TK-Renilla (1 ng per well) were co-transfected into the cells with Lipofectamine™ 3000 (L3000015, Thermo) according to the manufacturer instructions. After 48 h transfection, the cells were washed with PBS, and lysed in PLB (Promega), and the luciferase activity was detected according to the instructions for the Dual-Luciferase Reporter Assay System (Promega).

### Poly (I:C) assay

We used Poly (I:C) to mimic dsRNA derived from ERVs. *Setdb1* cKO mESCs were infected by lentivirus to overexpress Ripk3. Poly(I:C) (1 μg per well) was transfected twice at Day0 and Day2 using Lipofectamine™ RNAiMAX Transfection Reagent (13778150, Invitrogen) according to the manufacturer’s instructions. Poly(I:C) was generated by 1:1 mixing of Poly(I:C) HMW (tlrl-pic, InvivoGen) and Poly(I:C) LMW (tlrl-picw, InvivoGen).CCK8 assay was performed at day4.

### dCas9 assay

Pb-dCas9-Kdm4b-VP64 or pB-CRISPRoff was transfected into *Setdb1* cKO mESCs with Lipofectamine™ 3000. Cells were selected with Blasticidin for 2 weeks. sgRNAs targeting ERVs were constructed into the sgRNA backbone without Cas9. sgRNAs were transfected twice at Day0 and Day2 using Lipofectamine™ 3000. Cells was re-plated in 96-well plates at day 2 and CCK8 assay was performed according to the manufacturer’s instructions at day4.

### RNA-seq analysis

RNA-seq clean reads were mapped to mouse transcript annotation of Gencode vM15 version on mm10 genome using RSEM. We chose Trans Per Million (TPM) value for the normalization and evaluation of gene expression levels.

### ATAC-seq

ATAC-seq was performed as previously described [40]. Briefly, a total of 50,000 cells were washed once with 50 μl of cold PBS and resuspended in 50 μl lysis buffer (10 mM Tris-HCl pH 7.4, 10 mM NaCl, 3 mM MgCl2, 0.2% (v/v) IGEPAL CA-630). The suspension of nuclei was then centrifuged for 10 min at 500 g, 4 °C, and then 50 μl transposition reaction mix (10 μl 5 × TTBL, 5 μl TTE Mix V50 and 35 μl nuclease-free H2O) of TruePrep™ DNA Library Prep Kit V2 for Illumina® (TD501, Vazyme) was added. Samples were incubated at 37 °C for 30 min for PCR amplified. DNA was isolated using a MinElute® PCR Purification Kit (QIAGEN). ATAC-seq libraries were constructed using the TruePrep™ DNA Library Prep Kit V2 for Illumina® (TD501, Vazyme) and the library was then PCR amplified for the appropriate number of cycles. Libraries were purified with a MinElute ® PCR Purification Kit (QIAGEN). Library concentration was measured using VAHTS Library Quantification Kit for Illumina (NQ102, Vazyme). Finally, the ATAC library was sequenced on a NextSeq 500 using a NextSeq 500 High Output Kit v2 (150 cycles) (FC-404–2002, Illumina) according to the manufacturer’s instructions. All the sequencing data were aligned to the mouse genome assembly (mm10) using the Bowtie2 (version 2.2.5) with the options: -p 20 --very-sensitive --end-to-end --no-unal. Reads mapping to mitochondrial DNA or unassigned sequences were discarded. For pair-end sequence data, only concordantly aligned pairs were kept. Alignment bam files were transformed into read coverage files (bigwig format) using deep Tools with the RPKM (Reads Per Kilobase per Million mapped reads) normalization method.

### Quantification and statistical analysis

Data are presented as mean ± s.e.m. or mean ± s.d. as indicated in the figure legends. Unpaired two-tailed Student’s *t* test, Two-way AVOVA with Sidak’s multiple comparisons test were used to assess statistical significance. The *p* value, *t*-ratio were calculated with the Prism 8 software. A *p* < 0.05 was considered as being statistically significant, *p < 0.05, **p < 0.01, ***p < 0.001. No statistical method was used to predetermine sample size.

## Supplementary information

Supplementary Figure and legend

WB original data

supplementary information

checklist

## Data Availability

A BioProject accession number (PRJCA010501) has been assigned to the sequencing data of this manuscript. Other published datasets include PRJNA544540.
