# Supplementary material for: Transposable elements activation triggers necroptosis in mouse embryonic stem cells
Source: Cell Death Dis. 2023 Mar 7;14(3):184. doi: 10.1038/s41419-023-05705-3 (PMC9992707; doi:10.1038/s41419-023-05705-3)
Supplement: Supplementary file 1 — Supplementary Figure and legend [file 41419_2023_5705_MOESM1_ESM.docx]

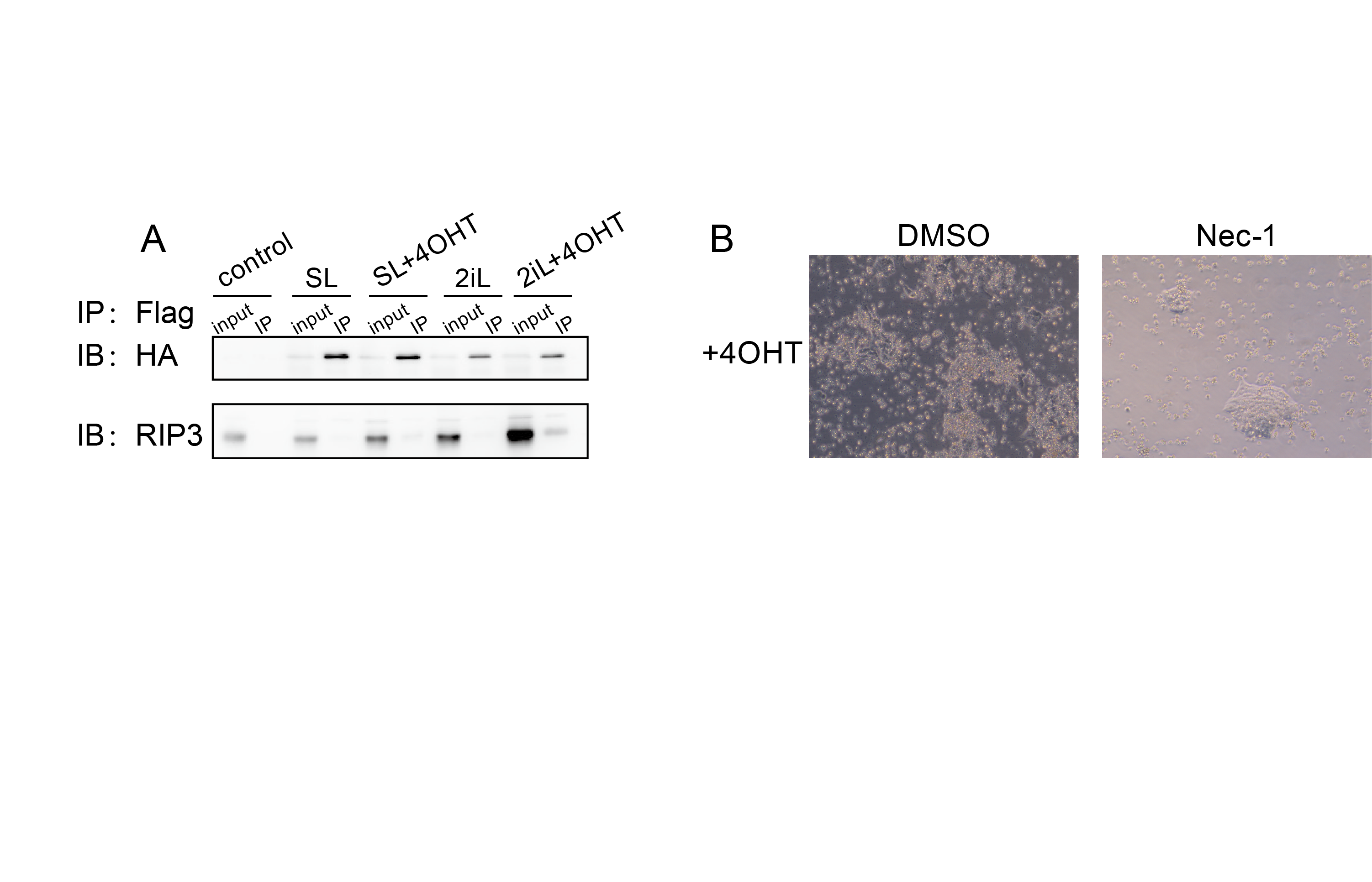


**Supplementary Figure 1 Setdb1 knockout induce necroptosis through RIPK1**. **A** co-IP of overexpressed HA-RIPK1 and RIPK3. **B** Nec-1 inhibits necroptosis induced by Setdb1-knockout.


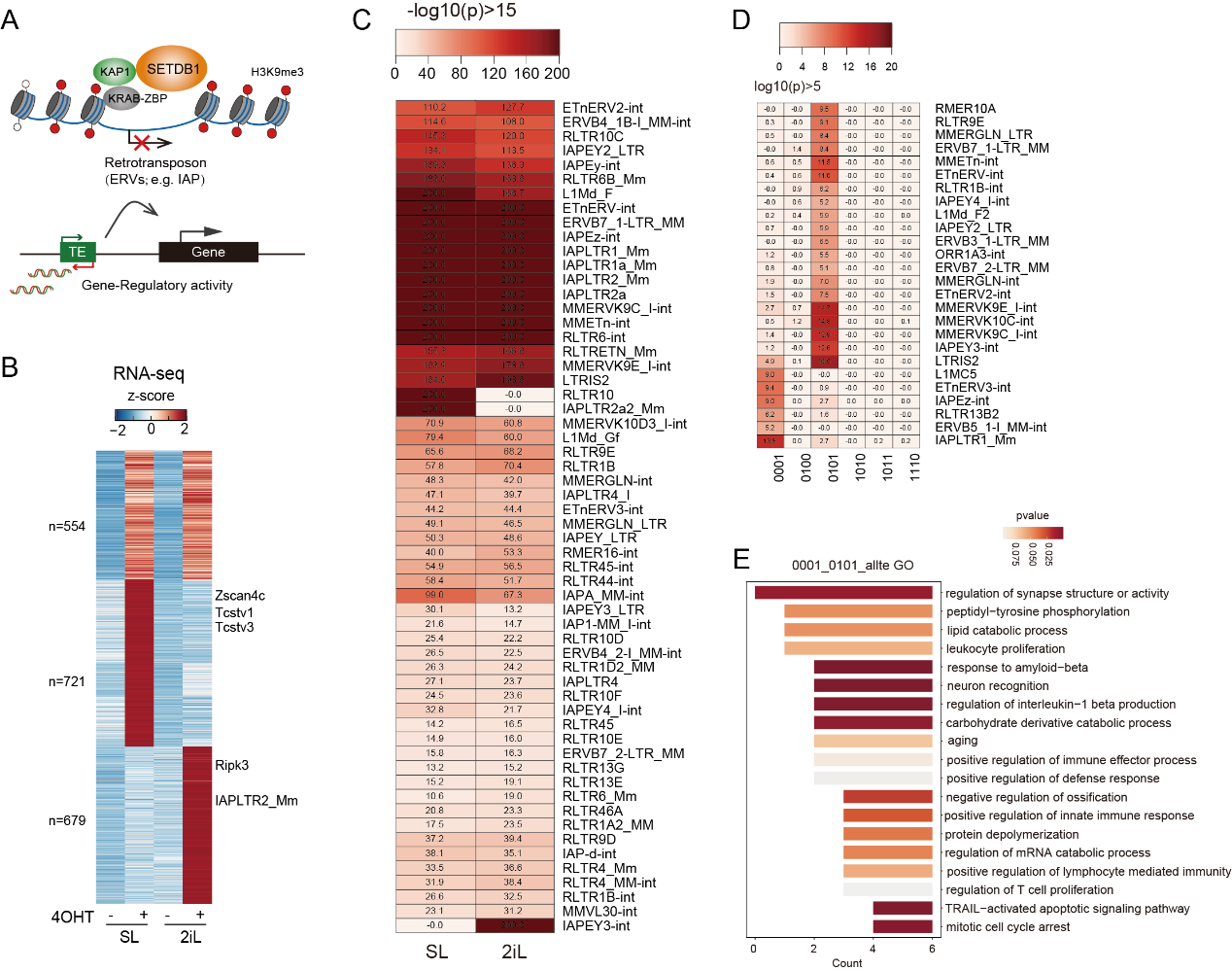


**Supplementary Figure 2 Depletion of *Setdb1* activates lots of TEs in SL and 2iL culturing conditions. A** Model of SETDB1 suppressing ERVs in mESCs. **B** Heatmap of RNA expression in SL and 2iL with/without 4OHT. **C** Heatmap of TEs expression in SL and 2iL culturing conditions. **D** Heatmap of TEs expression in cluster “0001”,”0100”,”0101”,”1010”,”1011” and “1110”. Significance is derived from a Fisher exact test. **E** GO analysis of cluster “0001” and cluster ”0101”. Significance is derived from a Fisher exact test.


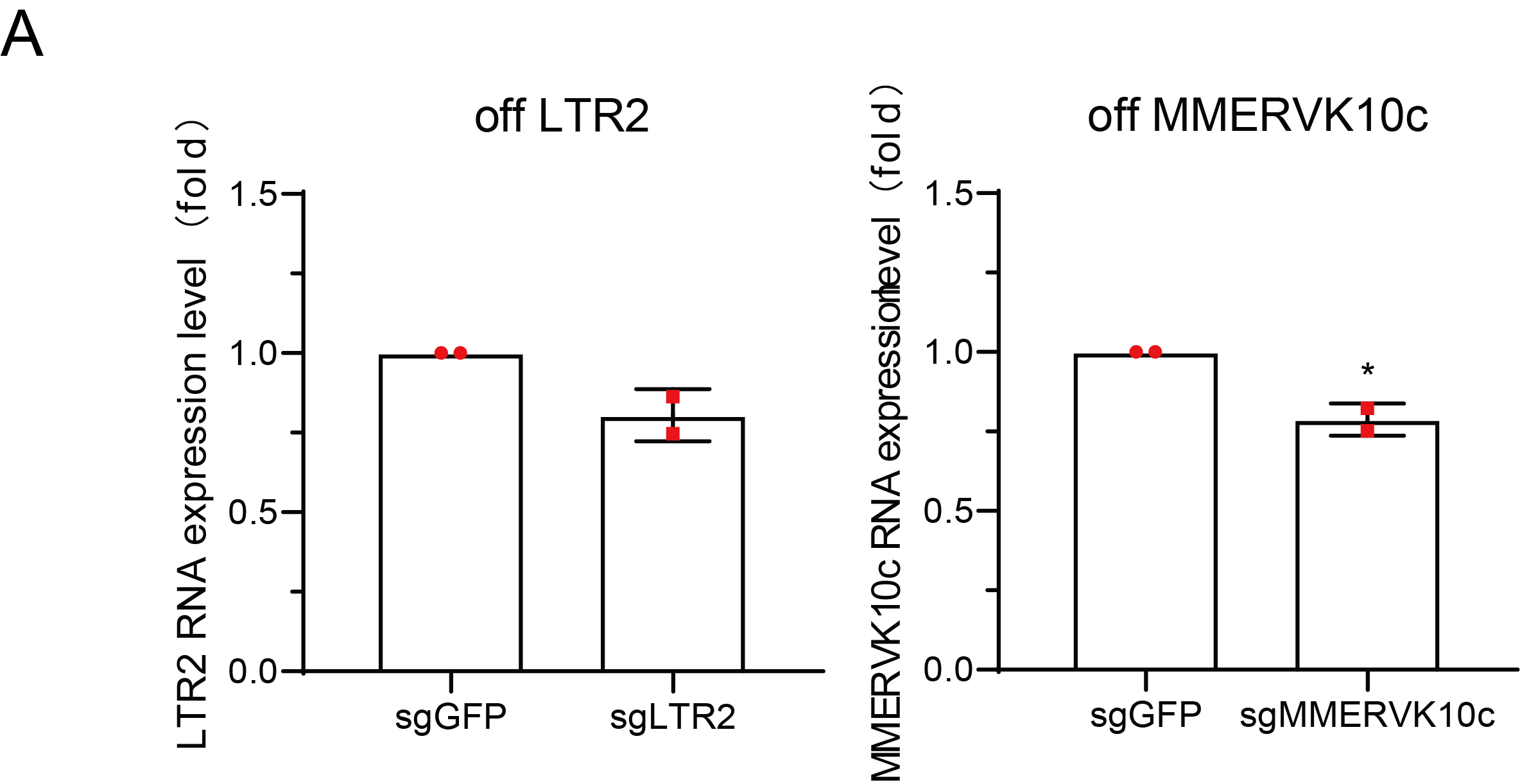


**Supplementary Figure 3A** Targeted TEs expression of *Setdb1* cKO cells overexpressing CRISPRoff. Cells were transfected with control sgRNA and sgRNA targeting IAPLTR2/MMERVK10c (n=2 biological replicates; error bar, SD; unpaired t-test, *p < 0.05, **p<0.01, ***p<0.001).


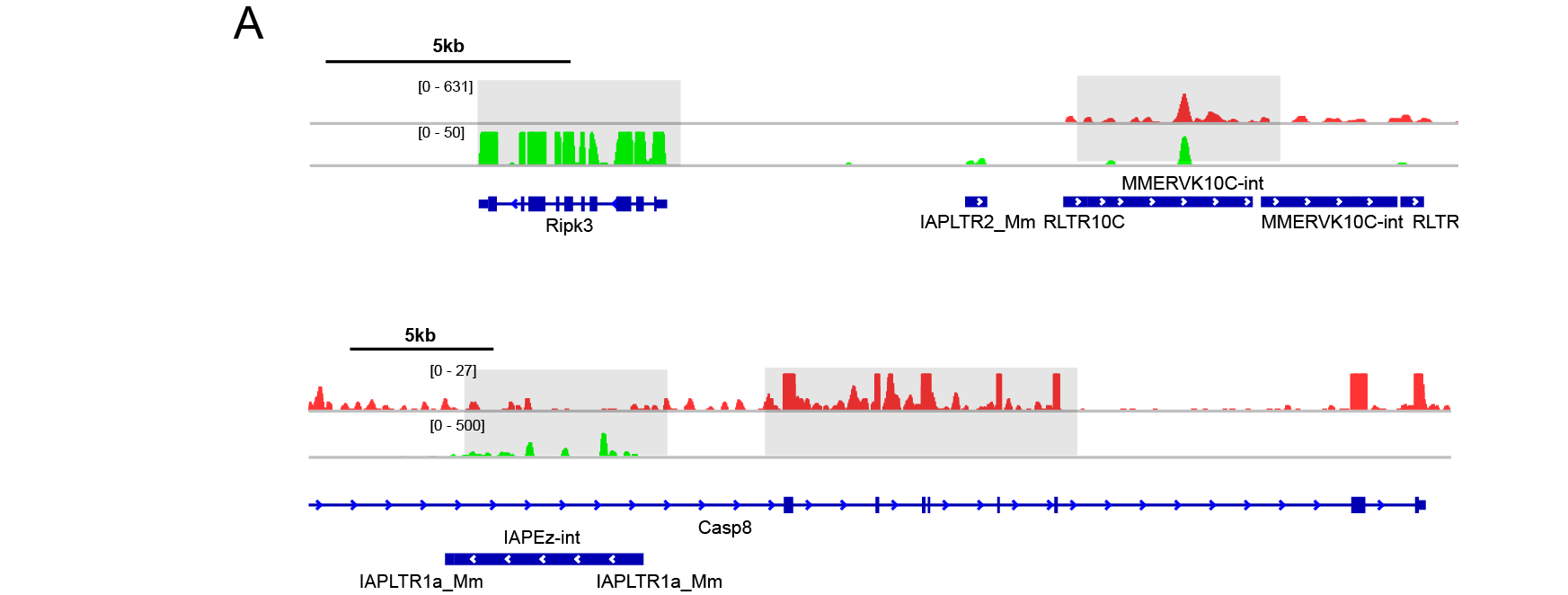


**Supplementary Figure 4A** IGV graphs illustrate peaks on ERVs loci and gene body in Setdb1 cKO cells with 4OHT treatment. ERVs peak exist in both direction while genes peaks only exist in one-way.


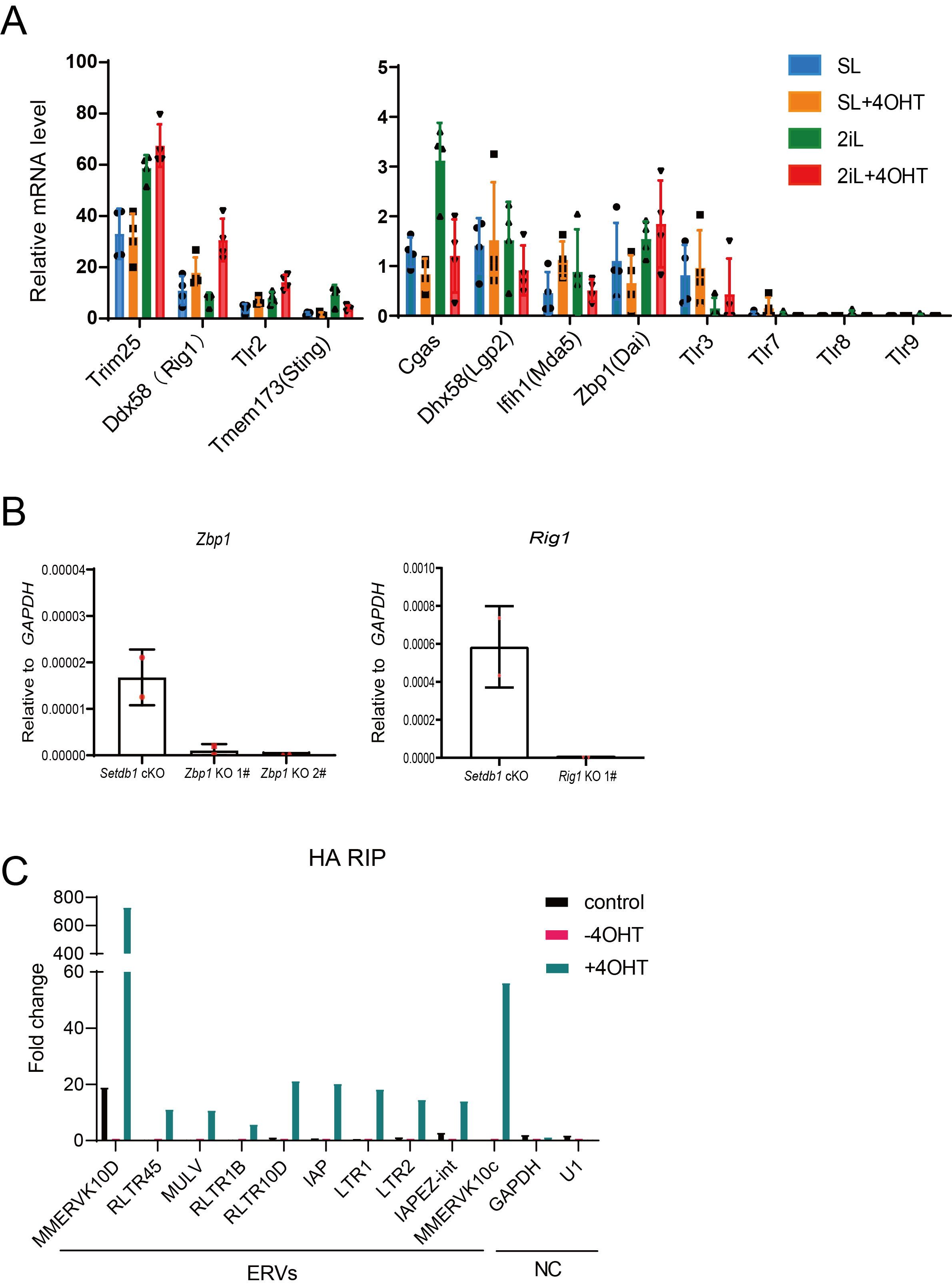


**Supplementary Figure 5 A** mRNA expression level of PRRs in SL and 2iL culturing conditions in Setdb1 WT/KO mESCs. **B** qPCR analysis demonstrates Zbp1 and Rig1 expression level in Setdb1 cKO cells after Zbp1 KO and Rig1 KO. (n=2 biological replicates; error bar, SD; unpaired t-test, *p < 0.05, **p<0.01, ***p<0.001). **C** HA-RIP qPCR shows that ZBP1 can bind several ERVs.
