# Supplementary figures and images for: Transposable elements activation triggers necroptosis in mouse embryonic stem cells
Source: Cell Death Dis. 2023 Mar 7;14(3):184. doi: 10.1038/s41419-023-05705-3 (PMC9992707; doi:10.1038/s41419-023-05705-3)

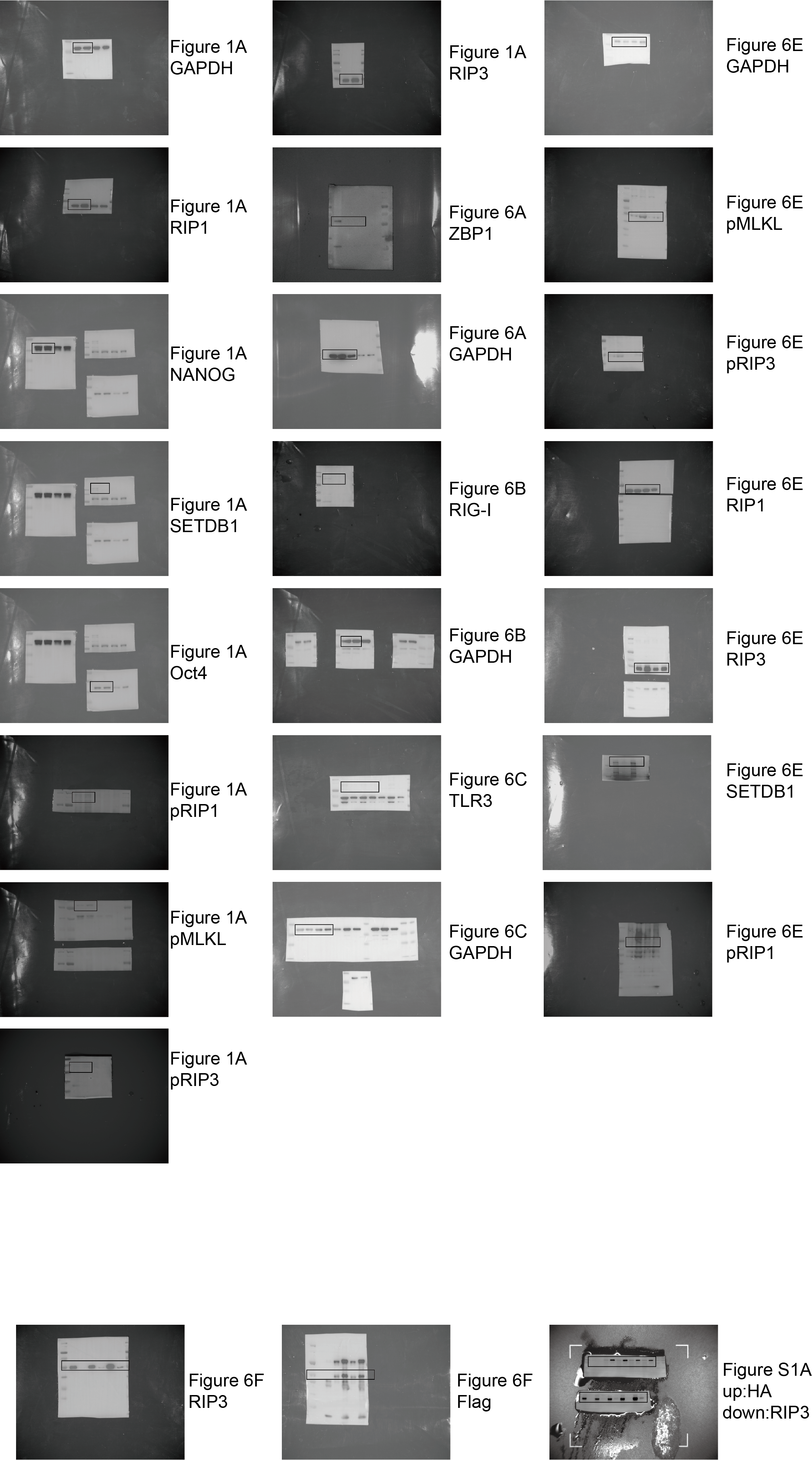

Supplement: Supplementary file 2 — WB original data [file 41419_2023_5705_MOESM2_ESM.png]
